# Recovery of horse fly populations in Louisiana marshes following the Deepwater Horizon oil spill

**DOI:** 10.1038/s41598-018-31442-1

**Published:** 2018-09-13

**Authors:** Claudia Husseneder, Jong-Seok Park, Lane D. Foil

**Affiliations:** 1 Department of Entomology, Louisiana State University Agricultural Center, Baton Rouge, LA 70803 USA; 2 S1-5 204b, Chungbuk National University, 1Chungdae-ro, Seowon-gu, Chengju, Chungbuk 28644 South Korea

## Abstract

The Deepwater Horizon oil spill in April 2010 had unprecedented impact on the Gulf of Mexico. We established the greenhead horse fly (*Tabanus nigrovittatus* Macquart) as a bioindicator of marsh health. This species is bound to coastal marshes, since its larvae develop as top invertebrate predators in the marsh soil. Immediately after the oil spill (2010–2011), populations of this horse fly declined in oiled areas of Louisiana marshes with significant impacts on genetic structure. In this follow-up study five years after the catastrophic event (2015–2016), we now report signs of recovery of populations in formerly oiled areas. Fly numbers increased compared to previous counts. Previously detected genetic bottlenecks in oiled populations have disappeared. Migration into oiled areas began to replenish formerly depleted horse fly populations in impacted regions with populations from non-oiled areas as an important source of migrants. Parameters of family structure that had been impacted by the oil spill (number of breeding parents, effective population size, number of family clusters) rebounded to levels similar to or exceeding those in non-oiled control areas.

## Introduction

In April 2010, the Deepwater Horizon (DWH) oil spill caused the largest man-made accidental marine oil spill to date with a total release of approximately 5 million barrels of oil into the Gulf of Mexico^1^ causing ecosystem-level injury to marine and coastal environments. Coastal Louisiana received the largest share of contamination by oil/dispersant mixture of all the Gulf States impacting its valuable and vulnerable salt marshes^2–6^.

When the oil made landfall, wide-spread destruction of marsh vegetation was observed^3,7,8^. Microbial and meiofaunal communities decreased in diversity and community function shifted towards oil degrading taxa^9–12^. The large majority of infauna that serve as prey for higher trophic levels (meiobenthos, nematodes, ostracods, copepods, annelids, among others) experienced high mortality in both heavily and moderately oiled sites in Louisiana salt marshes with their lowest diversity and density occurring 18 months after the oil spill^13–16^. Furthermore, McCall and Pennings^17^ reported that arthropod predators, herbivores, parasitoids and detrivores were suppressed by 50% at oiled sites in 2010.

One of the most extensive studies on the impact of the oil spill on an arthropod species with close association to the salt marsh was our study of population changes of the greenhead horse fly, *Tabanus nigrovittatus* Macquart (Diptera: Tabanidae), in oiled areas^18^. We established this horse fly species as our prime bioindicator model of marsh health for several reasons: 1. this species is native and linked to *Spartina* marshes from Texas to Nova Scotia; 2. the carnivorous larvae of this species develop for 3–9 months as the top predators within the invertebrate food web in the marsh soil, and their development is dependent on and a sign for the presence of a healthy food web in the sediment; 3. adult populations are highly visible, easy to catch, and 4. have shown immediate responses to the presence of oil in the marsh^18,19^. Population census and genetic studies of *T. nigrovittatus* showed population decline in both adult and larval numbers in oiled locations compared to unoiled locations in 2010 and 2011^18^. Genetic bottlenecks, loss of breeders, shrinking family sizes, and reduced migration rates provided combined proof for a loss of effective population size and a change in population structure in oiled areas. The immediate decline in the adult population was likely due to attraction to oil sheen on the water since oiled lakes have been shown to be insect traps^20^.

We presumed that the recovery of the greenhead horse fly would be closely linked to the decline of oil toxicity and subsequent recovery of infaunal communities reported in the literature and could be used as a measurable indicator for the underlying recovery of the *Spartina* marsh and the food web it contains. Therefore, we followed up on the fate of the greenhead horse fly in Louisiana *Spartina* marshes 5 to 6 years after the oil spill by pursuing the following scientific questions.

1. Have census numbers of adult horse flies in oiled areas increased in 2015 and 2016 compared to the years immediately after the oil spill (2010–2011)?
2. Have successful migrants replenished formerly depleted populations in oiled areas and has migration changed the population genetic structure over time?
3. Do the previously detected genetic bottlenecks in oiled populations still exist?
4. Have parameters of family structure that were impacted by the oil spill changed to indicate increased effective population size and population recovery when reexamined in 2015 and 2016?

## Results

### Signs of increases in abundance of horse fly adults and larvae in formerly oiled areas

**Figure 1:**
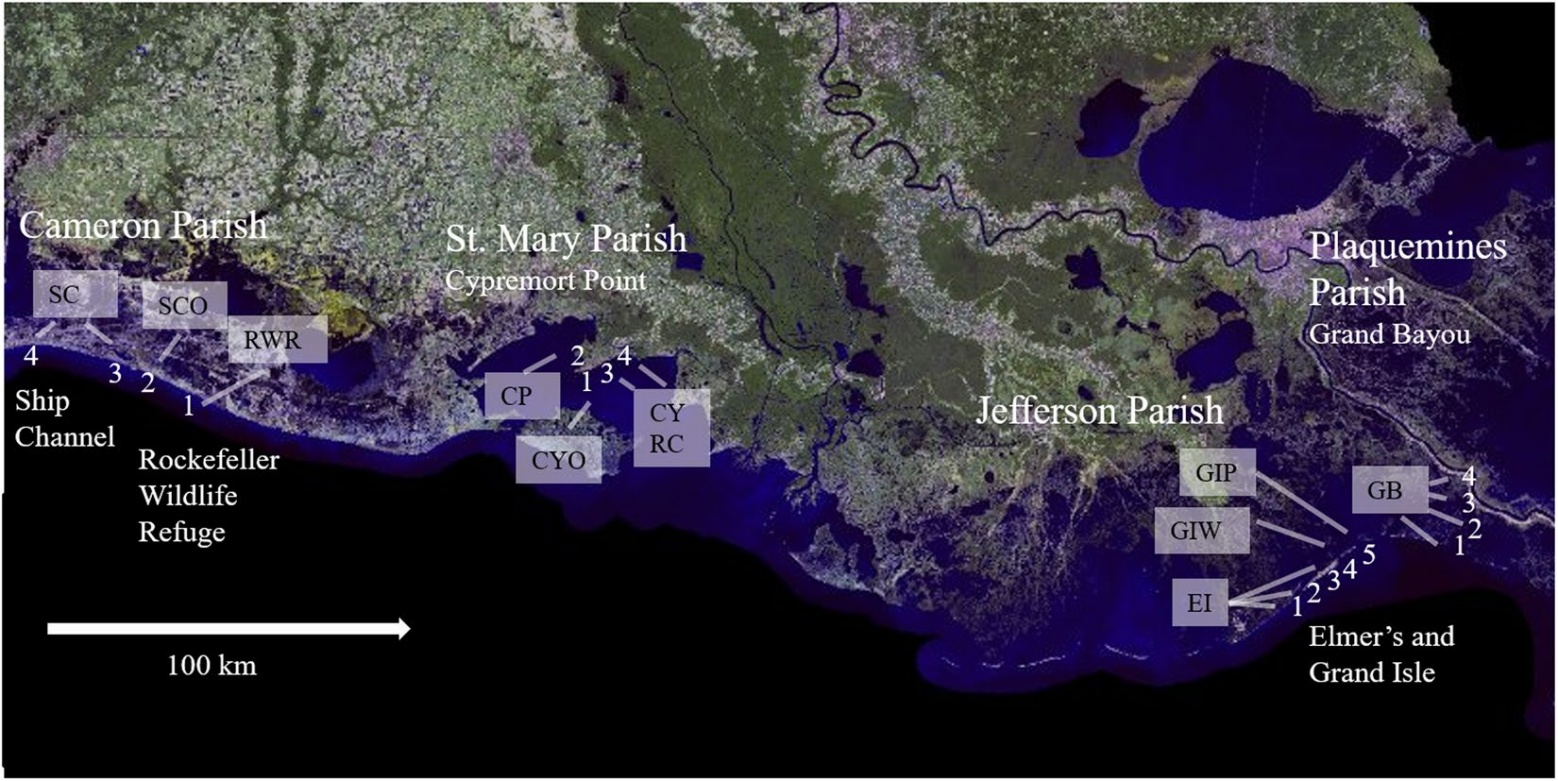
Map of the sampling locations of tabanid populations from non-oiled (Cameron and St. Mary Parish in West Louisiana) and oiled (Jefferson and Plaquemines Parish in East Louisiana) regions. Adult collections from four locations with 4–5 traps at each site were used for population abundance studies^18^. Samples also used for population genetic analyses are marked by text boxes (SC = Ship Channel, SCO = Oak Grove, RWR = Rockefeller Wildlife Refuge, CP = Cypremort Park, CYO = Cypremort Point, CYRC = Cypremort Road and Canal, EI = Elmer’s Isle, GIW = Grand Isle West, GIP = Grand Isle Park, GB = Grand Bayou).The background map is based on imagery published online by the United States Geological Service [https://cmgds.marine.usgs.gov/publications/of2008-1195/html/imagepages/land_sat.html].

Immediately after the oil spill (2010, 2011), adult horse fly trap catches were magnitudes lower in oiled areas; yet in 2016, numbers of adult horse flies caught near oiled areas in Plaquemines Parish were not significantly different from those caught in the non-oiled areas of Cameron Parish. In 2016, adult horse fly trap catches in formerly oiled areas, i.e., Elmer’s Isle, Grand Isle (Jefferson Parish) and Grand Bayou (Plaquemines Parish, Fig. 1) have increased (Table 1) compared to the severely reduced population numbers in oiled areas in 2010 and 2011. Although increases in adult numbers in formerly oiled areas were small, the incidence rate of detecting larvae in the marsh soil rose seven-fold at least in the Grand Bayou location. In 2011, we found only 1 larva in 8 sediment samples (0.13 larvae per sample) at Grand Bayou, while in 2016 we found 14 larvae in 16 samples (0.9 larvae per sample). The latter lies within the range of larva recovered from non-oiled areas with an average of 1–3 larvae per sample in 2011 and 0.8–1.2 larvae per site in 2016 at Cypremort Point and Rockefeller Wildlife Refuge. In contrast to the increased larval numbers at Grand Bayou, the larval count in 15 samples collected at Grand and Elmer’s Isle stayed zero in 2016, although slightly more adult flies were collected.

**Table 1:** Mean number of adult *Tabanus nigrovittatus* trapped (flies per hour) by region and year.

Region/Parish	2010		2011		2016	
	Mean ± SE	Mean (log x + 1) ± SE	Mean ± SE	Mean (log x + 1) ± SE	Mean ± SE	Mean (log x + 1) ± SE
Cameron	82.24 ± 6.62	3.88 ± 0.17^a^	53.25 ± 6.92	3.35 ± 0.18^a^	14.07 ± 3.09	2.39 ± 0.15^bc^
St. Mary	37.97 ± 6.64	2.62 ± 0.17^b^	38.01 ± 6.49	3.32 ± 0.17^ab^	55.61 ± 14.40	3.38 ± 0.26^a^
Jefferson	0.85 ± 6.06	0.42 ± 0.16^f^	1.34 ± 6.46	0.62 ± 0.17^ef^	1.72 ± 0.40	0.86 ± 0.09^ef^
Plaquemines	3.94 ± 5.82	1.17 ± 0.15^de^	4.51 ± 5.43	1.13 ± 0.14^def^	5.83 ± 0.97	1.73 ± 0.18^cd^

Different letters indicate statistical difference (P < 0.005, Tukey-Kramer).

### Population genetic structure

The five trap samples of adult tabanids from 2015 were all genetically different from each other based on their allele frequencies at the 5% level (10,000 permutations, FSTAT) determined by microsatellite genotyping. However, not all of the 18 trap samples (312 individuals) collected in 2016 were significantly genetically differentiated based on their allele frequencies at the 5% level (153,000 permutations, FSTAT). We combined samples that were not genetically differentiated and collected within the same location. This process resulted in eight genetically independent populations for 2016. For comparison, these genotyping data were analyzed together with data collected immediately after the oil spill^18^.

**Figure 2:**
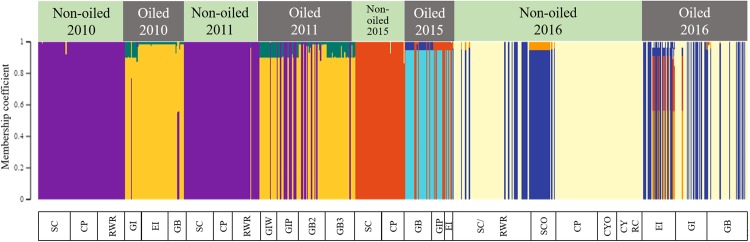
Tabanid population genetic structure from oiled and non-oiled regions of the Louisiana Gulf coast (2010–2016). The graph represents assignment of adult tabanid individuals from 26 populations (x-axis) collected from 2010 to 2016 from unaffected and oiled locations to eight major genetic clusters. The height of colored bars in each column represents the membership coefficient, i.e. the likelihood with which an individual is assigned to each genetic cluster. Number of genetic clusters (Kmax = 8) was determined using DIC curves (Fig. S1).

Population genetic analysis via TESS assigned the individual tabanids from the 26 sample populations spanning the years from immediately after the oil spill (2010 and 2011) to six years after (2015 and 2016) to eight major genetic clusters (Fig. S1 in Supporting Information). The plot of the membership coefficients (Fig. 2) that assign each individual horse fly to its predominant genetic cluster(s) visualizes the change in population genetic structure of *T. nigrovittatus* in oiled and non-oiled areas over a period of six years following the DWH oil spill.

The 2010 populations collected immediately after the oil spill of oiled and non-oiled areas fell into distinctly separated genetic clusters with none of the individuals assigned with membership coefficients of >80% to any region other than their own. In the first two years after the oil spill, the control populations were homogeneous suggesting high gene flow among those populations. In 2011, genetic signatures from the control populations (purple, Fig. 2) started to show up in the oiled regions indicating beginning migration from non-oiled to oiled regions, but not vice versa. Only one individual in one non-oiled population (RWR) showed affiliation to genetic signatures seen in oiled regions (gold, Fig. 2). In 2015, populations from formerly oiled regions showed still distinct genetic signatures with individuals belonging to genetic clusters not found in the control populations (cyan, Fig. 2). Similar to 2011, however, there was a considerable influx of genotypes typically found in the control areas (red, Fig. 2).

In 2016, essentially the same genetic signatures were found in individuals and populations from oiled and non-oiled regions (blue and cream, Fig. 2). Only some individuals from Elmer’s Isle (EI) retained a somewhat distinct genetic signature (red and orange, Fig. 2). Populations from oiled regions in 2016 showed a greater degree of heterogeneity, possibly reflecting immigration from different genetic clusters (blue and cream, Fig. 2) from the control populations.

### Genetic distances and migration rates

Genetic distances (*F*_ST_) among non-oiled populations showed no significant difference among the years (all P ≥ 0.10, two-tailed difference of means test, 300 permutations, Table S1). The genetic distances among oiled populations decreased over the years with distances in 2016 (*F*_ST_ = 0.083, SD = 0.064) being significantly lower compared to 2010 (*F*_ST_ = 0.259, SD = 0.032, P = 0.047) and 2011 (*F*_ST_ = 0.200, SD = 0.054, (P = 0.017). Distances were also lower (P = 0.037) in 2015 (*F*_ST_ = 0.129, SD = 0.113) than in 2010 and marginally lower (P = 0.097) in 2011 (Table S1). The genetic distances between populations from non-oiled vs. those from oiled regions, which were considerably high in the two years immediately after the oil spill, decreased after 2011 with 2016 *F*_ST_-values (0.163, SD = 0.095) being significantly lower than those from previous years (range 0.295 to 0.389, P < 0.001, Table S1).

Bayesian analyses demonstrated that recent migration rates (over the last few generations) among populations collected in 2015 and 2016 ranged from 0.1 to 25% with 68–99% being derived from the source populations in each generation (Table 2). This range is almost identical to what was recorded in 2010 and 2011^18^. However, there were changes in source and direction of migration patterns. Immediately after the spill event in 2010, only migration rates from and into non-oiled regions (see Table S4 in^18^) exceeded the minimum value required for informative levels obtained from simulations in BAYESASS^21^. None of the populations from oiled areas showed meaningful levels of migration rates in 2010. Gene flow among geographically close oiled areas connecting Grand Isle and Grand Bayou populations with migration rates ranging from 0.026 to 0.261, was first detected in 2011, one year after the spill^18^, but continued in 2015 and 2016 (Table 2).

**Table 2:** Proportions of migrants from and into each population.

2015 *0.0417	From	Non-oiled Regions					Oiled Regions		
		SC	CP				GB	GIP	EI
**Into**									
SC		*0.989*	0.003				0.003	0.003	0.003
CP		0.004	*0.986*				0.004	0.003	0.003
GB		0.006	0.006				*0.750*	**0.233**	0.006
GIP		0.013	**0.082**				0.012	*0.882*	0.011
EI		0.019	**0.119**				0.017	**0.147**	*0.698*
**2016** *0.0236	**From**	**SC/RWR**	**SCO**	**CP**	**CYO**	**CYRC**	**EI**	**GI**	**GB**
**Into**									
SC/RWR		*0.792*	0.011	**0.122**	0.002	0.004	0.002	**0.065**	0.002
SCO		**0.041**	*0.927*	0.014	0.004	0.004	0.003	0.004	0.003
CP		0.001	0.001	*0.992*	0.001	0.001	0.001	0.001	0.001
CYO		0.007	0.006	**0.245**	*0.684*	**0.040**	0.006	0.006	0.006
CYRC		0.005	0.003	**0.027**	0.003	*0.954*	0.003	0.003	0.003
EI		**0.030**	0.003	0.003	0.004	0.021	*0.778*	**0.149**	0.003
GI		**0.043**	0.007	**0.038**	0.004	**0.109**	0.012	*0.783*	0.004
GB		0.016	0.019	**0.075**	0.004	**0.148**	0.004	**0.060**	*0.674*

*Simulations were conducted for each data set to determine the value above which migration rate is informative (bold). Values in italics represent the proportion of individuals derived from the source population.

Migration from non-oiled into oiled areas and vice versa was mostly below the detection threshold in 2010 and 2011 except for one non-oiled western population (SC) that contributed small proportions of migrants (m) to oiled areas in the east (GIP: m = 0.017 and GB3: 0.073) in 2011^18^. In 2015, however, migration from non-oiled regions into formerly oiled regions increased with CP being the foremost source population (into GIP: m = 0.082, into EI: m = 0.119). In 2016, migration was observed from most of the non-oiled regions into all formerly oiled regions with reciprocity in one instance (from oiled GI into SC/RWR: 0.065, Table 2).

The average emigration rate from non-oiled regions almost doubled in 2015 and 2016 and was significantly higher than in the two years after the spill (Table S2). Emigration from oiled areas spiked in 2015. In 2016, emigration from oiled areas was still higher than immediately after the oil spill but the difference was not significant due to large variance among populations (Table S2). Immigration into non-oiled areas also showed considerable variance among populations and, thus, did not change significantly across the years, except for an increase in 2016 compared to 2011 (P = 0.01).

In 2016, immigration was significantly higher than in both 2010 (P = 0.001) and 2011 (P = 0.05, Table S2). Despite the small sample size in 2015, significantly elevated immigration rates were observed compared to 2010 (P = 0.002), but not to 2011 (P = 0.52). Immigration into oiled areas surpassed immigration into non-oiled areas in 2011 (P = 0.001), 2015 (P = 0.085) and 2016 (P = 0.002).

The main sources of repopulation from non-oiled into oiled areas shifted across the years. In 2010, no migration from non-oiled into oiled areas was detected. In 2011, SC showed minor contributions in terms of migrant proportions to GIP (m = 0.017) and GB2 (m = 0.073)^18^. In 2015, increasing proportions of migrants from CP were detected in GIP (m = 0.082) and EI (m = 0.119). Finally, in 2016, migrant proportions ranging from 0.03 to 0.14 originating from non-oiled areas (SC/RWR, CP, and CYRC) contributed to all formerly oiled populations (EI, GI, GB) (Table 2).

### Previously confirmed genetic bottlenecks in oiled populations disappeared five years after the oil spill

In contrast to populations collected immediately after the oil spill^18^, none of the populations collected in 2015 and 2016 showed signs of recent genetic bottlenecks. No heterozygote excess resulting from a population crash induced rapid loss of allele numbers was detected under the three mutation models (IAM, TPM, SMM, Table 3), regardless whether the area was oiled in 2010 or not. On the contrary, the majority of the populations, including those from oiled areas, showed significant homozygote excess in at least one of the mutation models.

**Table 3:** Probabilities to reject mutation-drift equilibrium due to heterozygote deficiency or heterozygote excess (genetic bottleneck) for three different mutation models (IAM = infinite allele model, TPM = two-phase mutation model, SMM = stepwise mutation model) in tabanid populations from unaffected and oiled areas.

	Non-oiled areas 2015		Non-oiled areas 2016					Oiled areas 2015			Oiled areas 2016		
	SC-2015	CP-2015	SC/RWR-2016	SCO-2016	CP-2016	CYO2016	CYRC-2016	GI-2015	EI-2015	GB-2015	GI-2016	EI-2016	GB-2016
**One-tailed Wilcoxon-test**													
**for heterozygote deficiency**													
IAM	>0.20	>0.20	>0.20	>0.20	0.004	>0.20	>0.20	>0.20	>0.20	>0.20	0.065	>0.20	>0.20
TPM	>0.20	>0.20	0.041	>0.20	0.008	>0.20	0.008	>0.20	>0.20	>0.20	>0.20	>0.20	>0.20
SMM	>0.20	0.03	0.006	0.037	0.008	>0.20	0.008	0.15	0.16	>0.20	0.002	0.016	0.02
**for heterozygote excess**													
IAM	>0.20	>0.20	>0.20	>0.20	>0.20	>0.20	>0.20	>0.20	>0.20	0.16	>0.20	0.08	>0.20
TPM	>0.20	>0.20	>0.20	>0.20	>0.20	>0.20	>0.20	>0.20	>0.20	>0.20	>0.20	>0.20	>0.20
SMM	>0.20	>0.20	>0.20	>0.20	>0.20	>0.20	>0.20	>0.20	>0.20	>0.20	>0.20	>0.20	>0.20

None of the populations showed genetic bottlenecks in 2015 or 2016 regardless whether the area was oiled in 2010 or remained unaffected. Note, that in 2010/2011 6 out of 7 populations from oiled locations showed signatures of genetic bottlenecks^18^.

### Comparison of family structure between populations from non-oiled and oiled areas collected across six years

Year had no significant effect in the previous data sets from 2010/2011 (GLM)^18^. The data sets were thus combined and included for comparison to the situation in 2015 and 2016 to allow assessment of recovery. “Year” had a marginal effect in 2015 and 2016 on the number of parents (P = 0.089, df = 1, F = 3.628) with significant interactions of “year” and “condition” on the number of parents (P = 0.006, df = 1, F = 12.789) and percent of half-sibs (P = 0.025, df = 1, F = 7.439, GLM) and the years were, thus, treated separately. When 2015 and 2016 data sets were added to the 2010/2011 data, “year” had significant effects on the number of parents (P = 0.012, df = 2, F = 5.546), the number of family clusters (P = 0.004, df = 2, F = 7.243), the percentage of half-sibs (P = 0.03, df = 2, F = 4.195), and a marginal effect on the effective population size (P = 0.077, df = 2, F = 2.915). “Condition” had a significant effect over all years on effective population size (P = 0.038, df = 1, F = 4.935), and the number of parents (P < 0.001, df = 1, F = 17.417).

**Figure 3:**
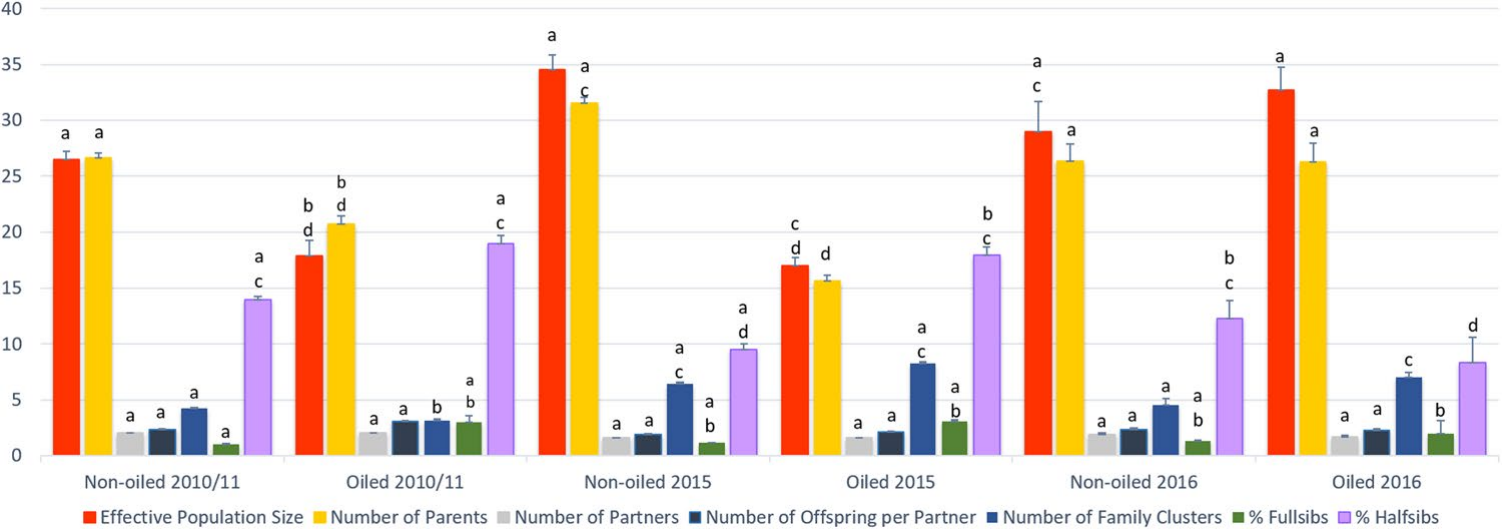
Parameters of population genetics and family structure of tabanids collected from oiled and non-oiled areas immediately after the oil spill to six years later. Shared letters in letter combinations above bars denote lack of significant difference (P > 0.05, two-tailed t-tests, SPSS) when comparing the same parameter measured in populations from oiled vs. non-oiled regions across the years. Significant differences (P ≤ 0.05) are indicated by unique letters.

Effective population size and number of parents contributing offspring to population samples were equally high in non-oiled areas across all years (Fig. 3). In the years immediately after the oil spill, the effective population size and the number of parents were significantly lower in oiled areas, consistent with the decline in adult populations (details and P-values in^18^). These differences between non-oiled and oiled regions persisted in the year 2015 (effective population size: P = 0.044, t = 3.346, df = 3; number of parents: P = 0.040, t = 3.465, df = 3). However, in 2016 both effective population size and number of parents had increased significantly in formerly oiled areas compared to 2010/2011 (effective population size: P = 0.002 t = −4.341 df = 8; number of parents: P = 0.05, t = −1.917, df = 8) and 2015 (effective population size: P = 0.005, t = −5.618, df = 4; number of parents: P = 0.033, t = −3.211, df = 4) and reached a level that is not significantly different from those of non-oiled areas across the years (P > 0.20).

The number of family clusters increased significantly in oiled regions in 2015 (P = 0.019, t = −2.938, df = 8) and 2016 (P = 0.001, t = −5.499, df = 8) compared to the number in oiled areas in 2010/2011. In 2015, the number of family clusters in oiled areas was not significantly different from the numbers in non-oiled areas anymore. Moreover, in 2016 the number of family clusters in formerly oiled areas became significantly higher than in control areas (P = 0.021, t = −2.954, df = 7).

Both sexes of *T. nigrovittatus* can be polygamous^18^. The number of partners each individual mated with and the number of offspring per partner did not differ across the years or between oiled and non-oiled regions. The percentage of full-sib pairs found in the sample populations was only different between non-oiled areas immediately after the oil spill and oiled areas in 2016. The percentage of half-sibs was significantly lower in oiled areas in 2016 compared to most other samples (except non-oiled 2015). In particular, the percentage of half-sibs was reduced in oiled areas sampled in 2016 compared to oiled areas in 2010/2011 (P = 0.019, t = 2.972, df = 8) and 2015 (P = 0.001, t = 9.568, df = 4).

## Discussion

A holistic understanding of the progression of long-term effects of oil spills as well as the timelines for recovery of salt marshes is essential for assessing the resilience of the marshes’ vital ecosystem services and fundamental ecological patterns and processes. Native infauna, i.e., the meio- and macrofauna developing in the salt marsh soil, are postulated to be the most informative indicators of ecosystem recovery after oil spills due to their important interactions as part of the food web. Here we discuss the observed recovery of the greenhead horse fly 5–6 years after the Deepwater Horizon oil spill in the framework of the multi-layered recovery of infauna with different life history, diversity and function.

We postulated that species native to the marsh, like *T. nigrovittatus*, with carnivorous sediment dwelling developmental stages placed at the top of the invertebrate food chain, should be most sensitive^22^ and, thus, most valuable bioindicators of marsh health. The DWH oil spill caused a severe drop in the numbers of horse fly larvae and adults in oiled areas, accompanied by underlying changes in population genetic and family structure^18^. This led to the question if, when, and how the process of recovery might mitigate or even restore the genetic make-up of tabanid populations colonizing or residing in oiled areas.

Six years after the oil spill, adult census numbers and the frequency of larvae being found in the soil were increased in formerly oiled areas compared to our findings immediately after the oil spill. Adult numbers as stand-alone, however, should be interpreted with caution. Census data often overestimate effective population size^23^. Trap catches vary due to seasonal fluctuations^18^, weather conditions and the occasional presence of horse fly predators such as dragonflies and bembix sand wasps, which cause horse flies to avoid traps^24^. Although increases in adult numbers in formerly oiled areas were small, the incidence rate of detecting larvae in the marsh soil clearly increased. The larva numbers (0.9 larvae per sample) in the formerly oiled Grand Bayou location reached the range of larva recovered from control areas. Since the larvae are cannibalistic, finding approximately one per sample would be expected in a productive habitat. The other formerly oiled areas (Elmer’s Isle and Grand Isle) were heavily impacted by clean-up and reconstruction efforts, which may explain why the ecosystem has not yet rebounded to sustain peak larval development. Although there were no pre-event data to know the “normal” baseline number of horse flies in specific areas before the oiling, the rising census numbers in formerly oiled areas could be interpreted as the first sign of population recovery, especially since recovery subsequently was confirmed six years after the spill by genetic measures.

The observed increase in effective population size, number of breeders and number of family clusters in formerly oiled areas, which reached the level of control populations in 2016, supported the rise in census numbers and explains the mitigation of genetic bottlenecks recorded after the oil spill. The quick (≤5 years) recovery from bottlenecks of tabanid populations from oiled areas is expected to reduce negative consequences on evolutionary potential and resilience of the impacted populations^25,26^ that are typically predicted and observed in slow recovering populations^27,28^. Most of the populations of 2015 and 2016 showed significant homozygote excess in at least one of the mutation models. While inbreeding is part of the life history of *T. nigrovittatus*^19^, the novel rise of heterozygote deficiency in populations of the formerly oiled areas likely indicates a reverse bottleneck effect, i.e., a recent population expansion. Population expansion in formerly oiled areas could be caused by 1. successful migration into oiled areas, 2. sufficient rate of reproduction in oiled areas to overcome residual larval mortality, if any, and 3. increased survival of larvae developing in formerly oiled areas due to decreased oil residue toxicity to larvae and/or increased availability of their food web.

1. In terms of migration capacity, tabanids rank as strong fliers and can disperse across considerable distances^18,29,30^. In the present study we recorded shrinking genetic distance (*F*_ST_) over the years between populations from non-oiled and oiled areas and among populations from oiled areas, which suggests increasing migration rates compared to the low gene flow among populations after the oil spill. Therefore, we tested the hypothesis that populations from non-oiled areas were an important source for repopulation of formerly depleted oiled areas.

The lack of migration of a typically strong flyer immediately after the oil spill in 2010 could be explained by reduced dispersal capacity of tabanids from oiled areas (comparable to the reduced swimming performance in fish after sublethal oil exposure^31,32^; and/or mortality of migrants into oiled areas, because flies were attracted by polarized reflections from oil sheens while searching for fresh water^33,34^. However, in 2011 (one year after the spill), some degree of migration was detected among geographically close oiled areas (Grand Isle and Grand Bayou) suggesting sufficient reduction of oil in the water and/or in the sediment to sustain survival of migrants. Still, larvae were almost entirely absent from formerly oiled areas in 2011^18^. The most important parameter in terms of recovery was the significant increase in migration from non-oiled areas into oiled areas over the years with 2016 showing migration from most of the non-oiled regions into all formerly oiled regions and even occasional contributions of migrants from oiled areas into non-oiled areas. This flow of migrants from populations of non-oiled regions (or closely related unsampled populations with the same genetic signature) into oiled areas was reflected in the increasing homogenization of population structure in the later years, which stands in contrast to the distinct genetic signatures of populations immediately after the oil spill in 2010.

These results highlight dispersal ability as an important driver of recovery. As a general rule, populations of organisms with rapidly dispersing life stages were the first to recover as soon as oil contamination and soil conditions were sufficiently improved to sustain survival. Similar patterns were also found in long-term colonization and succession studies of invertebrates in newly constructed salt marshes^35^. Taxa with dispersing larval stages, e.g. some annelids, copepods and nematodes, quickly colonized newly created marshes or re-colonized formerly oiled sites within three to five years to a level equivalent to healthy reference sites^16,35,36^. In contrast, slow recovery (>4 years) was shown for taxa that lack larval dispersal and those whose later stages are also poor dispersers due to their association with the marsh sediment^16,35^.

In comparison to these macro- and meiofauna invertebrates that are similarly native and tightly bound to the marshes as *T. nigrovittatus*, the dispersal and recovery of the latter was quicker than expected despite the fact that this tabanid has sediment-dwelling carnivorous (top of the food chain) larvae. The larvae are accomplished swimmers and can be observed hunting in the tidal zone, which likely facilitates dispersal at the larval stage to some degree (LF personal observation, https://www.youtube.com/watch?v=GAdm_JqAuoM). In addition, adults are excellent fliers, which explains the observed gene flow from unoiled into oiled areas as soon as one year after the spill.

2. While dispersal capacity was certainly a strong factor to facilitate population recovery of *T. nigrovittatus*, successful recolonization of formerly oiled areas also requires reproductive success. Invertebrates with slow recovery include the tube-dwelling oligochaetes, polychaetes and tanaids, whose relatively small offspring numbers develop confined within the maternal tubes^37^. In contrast, quick recovering invertebrates (annelids, nematodes, and copepods) produce and release high numbers of progeny into the water column^16^. *Tabanus nigrovittatus* is known to lay >200 eggs per batch and is an autogenous species, i.e., females can produce the first batch without the need for a blood meal and produce subsequent batches after blood meals have been obtained. The pursuit of a blood meal and subsequent oviposition would ensure dispersal of individuals and recovery of depleted areas. Genetic data suggest that males and females might be able to mate multiple times (polygamy) and have multiple offspring with each partner^18^. The high reproductive and dispersal capacity of tabanids should translate into rapid population growth and re-colonization of formerly oiled areas, but only if larvae can develop in the sediment. The observed rebound in effective population size of adults, number of successful breeders and families in formerly oiled areas required not only a certain degree of decontamination of formerly oiled sediments, but also sufficient secondary (heterotrophic) production and food web support for larval development.

3. The process of recovery of the food web had started bottom-up almost immediately after the oil spill setting the stage for the return of horse flies to oiled marsh regions. Microbial bioremediation of oiled marsh sites^9,10,38^ paved the way for vegetation recovery. Even in heavily oiled areas with near complete plant mortality initially, some recovery of was reported six years after the spill albeit total live aboveground biomass was <50% of reference marshes. In moderately oiled areas above ground vegetation recovered within 2.5 years^3,39^. Most microalgae and 90% of meiofauna (e.g., nematodes, copepods and most annelids), which form the base of the food web in the marsh sediments, recovered within three years after the DWH oil spill following closely the re-growth of *Spartina* marshes except for certain polychaetes, amphipods, kinorhynchs, ostracods and gastropods, that had not reached levels of reference marshes after 4 years^15,16^. According to McCall and Pennings’ study^17^, the terrestrial arthropod community, including mostly herbivorous insects, and intertidal crabs had largely recovered one year after the spill despite suppression by oil exposure a year earlier. The guts of tabanid larvae contain mostly insect DNA as revealed by a recent 18S metagenome sequencing study (unpublished data). The quick return of insects to oiled marshes was, therefore, vital for the tabanid recovery.

*Tabanus nigrovittatus* populations’ sensitivity to oil contamination evidenced by the immediate population crash following landfall of DWH oil^18^ and their recovery following in succession with regrowth of *Spartina* and recovery of 90% of the meiofauna^16^ makes this species a valuable bioindicator to assess the process of marsh reconstitution (Table 4). To provide more detail about what is required for the greenhead horse fly to colonize newly established wetlands or recolonize marshes after a catastrophic event, we are currently describing the food web of *T. nigrovittatus* larvae via 18S rRNA gene metagenomic sequencing of larval gut contents.

**Table 4:** Comparison of population parameters of tabanids collected from oiled and non-oiled areas across a period of six years after the oil spill.

	2010/2011		2015		2016	
	Not oiled	Oiled	Not oiled	Oiled	Not oiled	Oiled
Adult fly counts	High	Low	n/a	n/a	High	Rising
Larvae recovered from marsh soil	High	Low	n/a	n/a	High	Rising
Effective population size	High	Low	High	Low	High	High
Number of breeders	High	Low	High	Low	High	High
Number of families	High	Low	High	High	High	High
Number of migrants/gene flow	High	Low	High	Rising	High	High
Genetic bottlenecks	No	Yes	No	No	No	No

Data obtained immediately after the oil spill^18^ were included for comparison. n/a: survey data not collected in 2015.

## Material and Methods

### Collections

For population census data, adult female flies were collected using canopy traps baited with dry ice^40^ every other week from April through October in 2016, at the same four locations with 4–5 traps per site as described before (Fig. 1)^18^. Collection permits for horse fly larvae and adults were obtained from the Louisiana Department of Wildlife and Fisheries (permit number LNHP-16–060). The sites were selected relative to available access, personal observation (LF) of high horse fly abundance prior to the oil spill, absence of confined livestock, and vicinity to *Spartina* marshes. Cameron Parish and Cypremort Point in St Mary’s Parish in West Louisiana were not impacted by the oil spill (non-oiled controls). However, oiling was reported at Elmer’s and Grand Isle on May 24, 2010 and at Grand Bayou on June 18, 2010 [http://www.nytimes.com/interactive/2010/05/01/us/20100501-oil-spill-tracker.html, date of access: 1/26/2018]. The exact daily trapping period (ranging from 2–8 hours) was recorded for each site at each location, and the highest three trap counts were used for analysis. The captured flies were transferred on dry ice and stored at −80 °C. Specimens were identified as *T. nigrovittatus* and counted using a dissecting microscope and cold plate, and returned to −80 °C for storage. Voucher specimens were deposited in the Louisiana State Arthropod Museum at the Department of Entomology of the Louisiana State University Agricultural Center.

A set of five and 18 samples collected in June 2015 and June 2016, respectively, were used for population genetic studies and combined with the previously published data set^18^ for longitudinal comparison. For the data to be comparable to our microsatellite data set from 2010 and 2011, we genotyped horse flies that were collected during the same month (June) in 2015 and 2016. Samples were taken from single traps representing three different trap sites in the non-oiled marshland along the coast of Western Louisiana (Ship Channel, Rockefeller Wildlife Refuge, and Cypremort Point) and three trap sites from regions in Eastern Louisiana (Grand and Elmer’s Isle and Grand Bayou, Fig. 1) that were oiled or where oiling was reported in close proximity.

We also compared the presence or absence of tabanid larvae from 15–16 *Spartina* marsh substrate samples per location collected using the techniques of Dukes *et al*.^41^ on four occasions between 4/11/2016 and 8/2/2016 to the larval numbers sampled in 2011 at of the same four locations, i.e., Rockefeller Wildlife Refuge, Cypremort Point, Grand and Elmer’s Isle, and Grand Bayou^18^. Marsh samples were collected near adult collection sites by excavating 5 m long 0.5 m wide 10 cm deep transects. Tabanid larvae were removed from the sediment samples using a brine-flotation system, and the collected larvae were stored in 95% ethanol.

### Microsatellite genotyping

Total DNA was extracted from thoraces of up to 30 adult female horse flies per sample (trap) using the DNeasy Tissue Kit (Qiagen Inc., Valencia, CA) and genotyped using the Li-Cor 4300 automated DNA analyzer (Li-Cor Inc., Lincoln, NE) as described in Husseneder *et al*.^19^. The same microsatellite loci developed and used in previous population genetic studies of the greenhead horse fly^18,19^ were screened as described in^19^ before being employed to compare population genetic parameters in 2015 and 2016 to those measured after the oil spill (2010, 2011)^18^. Since a formerly monomorphic locus (7FA)^19^ showed multiple alleles in several samples of 2016, the number of polymorphic loci was increased from 10 (2010, 2011, 2015) to 11 (2016). Locus characteristics, GenBank accession numbers, and other summary statistics have been published previously^18,19^.

### Statistical analysis of adult horse fly abundance

Exploratory analyses indicated that data were not normally distributed. The average numbers in each trap collection were transformed to y = log(x + 1) to normalize prior to statistical analysis. A two-way ANOVA (PROC MIXED, SAS Institute 2010) was used to compare log transformed tabanid catch data across years and locations. Model main effects were Year (2010, 2011, 2016) and Region (Plaquemines, Jefferson, St. Mary, and Cameron). All possible interactions among the main effects in the model, and a Tukey-Kramer adjustment (α = 0.05) were used to separate the means. The catch data for 2010 and 2011 were published in Husseneder *et al*.^18^, but included for comparison to adult tabanid counts obtained six years after the oil spill.

### General population genetic statistics

Samples from 5 traps in June 2015 and 18 traps in June 2016 were tested for significant genotypic differentiation using pairwise log-likelihood *G*-Statistics with standard Bonferroni corrections at a 0.001 nominal level for multiple comparisons (FSTAT)^42^ and genetically separated samples (populations) were then used as population genetic units of analysis. Genetic distances among populations (*F*_ST_) were calculated using the methods of Weir and Cockerham (1984) in FSTAT. For comparison of pairwise distances two-tailed difference of means tests were performed with resampling techniques (300 permutations) using a modified Excel worksheet available at woodm.myweb.port.ac.uk/nms/diffofmeansconfidence.xls.

### Population genetic structure

The data set from 2010 and 2011^18^ was combined with the genotypes obtained for 2015 and 2016 to allow for longitudinal comparisons to detect changes in population genetic structure over time. For ad hoc approximations of the number of genetic clusters in the total population (2010–2016) and the genetic relationships among the 26 populations, individual flies were probabilistically assigned to genetic clusters based on their multilocus genotypes using TESS 3.1^43^. For exploratory analysis, the simulations were run 10 times with and without admixture for each Kmax ranging from 2–26 for 1,200 sweeps with 200 sweeps burn-in. The maximum number of genetic clusters (Kmax) was determined from changes in the deviance information criterion (DIC, Fig. S1)^44^. Since Kmax and cluster patterns were similar with both ancestry models, and population differentiation in previous studies made it less likely that the majority of individuals have recent ancestors from different populations, a full analysis was completed with 100 runs with 50,000 sweeps and 10,000 burn-in using the no admixture model. The 20 runs with the lowest DIC (20% filter) were averaged using CLUMPP 1.1.2^45^. Finally, the estimated membership coefficients of each individual multilocus genotype to each genetic cluster were plotted with STRUCTURE PLOT Version 2^46^.

### Migration rates

Bayesian statistics were employed (BayesASS v. 1.3) to estimate the proportion of migrants (Table 2) and immigration and emigration rates (Table S2), because these techniques allow for deviation from migration-drift balance and Hardy-Weinberg equilibrium^21^. Preliminary runs were used to assure that delta values for allele frequency, migration rate and inbreeding stayed between 20–60% of the total chain length^21^. The final settings were 3,000,000 iterations, a sample frequency of 2000, a burn-in period of 999,999 and delta values of 0.25.

### Detection of genetic bottlenecks

Immediately after a genetic bottleneck, allele numbers are reduced faster than heterozygosity. To detect signs of bottlenecks, genotypes of individuals in each population were tested for heterozygote excess across loci using a Wilcoxon sign-rank test under different mutation models (IAM, i.e., infinite allele model, TPM, i.e., two-phased model of mutation with 80% single step mutations and 20% multi-step mutations, SMM, i.e., stepwise mutation model), as implemented in BOTTLENECK v. 1.2.02^47^. In general, SMM and TPM are recommended when using microsatellite data since they are taking evolutionary relationships among alleles into account^47^. However, in our study over a limited geographical and time scale migration likely exceeded mutation as source of genetic variation and, therefore, we also presented results for the IAM, an implicit model that makes no assumptions on ancestry of alleles and origin of diversity.

### Mating structure and effective population sizes

Pedigree structure in each population was inferred using the full pedigree likelihood method implemented in COLONY v.2.0.3.1^48^. Program choice and parameter settings are described in detail in Husseneder *et al*.^18^. To infer the best configuration of relationship structure, populations were subjected to full maximum likelihood calculations^48,49^. Based on the Best Maximum Likelihood Configuration output of COLONY, we determined the number of inferred parents contributing offspring to each population (i.e., a measure of the breeding population size) and the number of family clusters in each population. Values were corrected for uneven sample sizes by calculating each for a sample size of 30 individuals genotyped per population. We also estimated the number of partners that individuals mated with and the number of offspring produced per parent. The percentages of full-sib and half-sib pairs in each population were inferred from sibship assignment plots^48^. Effective population size (*N*_e_) was inferred with the sibship assignment method in COLONY^48^. The sibship assignment method showed superior accuracy for “real-world” data sets compared to other methods since it does not assume isolated populations or random mating and performs comparatively well even with small sample sizes and numbers of loci^50^.

General Linear Model (GLM) analyses were used to test for effects of “year” and “condition” (oiled vs non-oiled) on the variables of mating structure and population size. For each variable pairwise comparisons were performed between populations from oiled and non-oiled regions and between each year using two-tailed t-tests (SPSS, IBM Corp. Released 2012. IBM SPSS Statistics for Windows, Version 21.0. Armonk, NY: IBM Corp.). *P*-values ≤ 0.05 were considered significant and *P*-values up to 0.10 were considered marginal.

### Metadata

Metadata are archived and available through the GRIIDC site of the Gulf of Mexico Research Initiative Information and Data Cooperative. Detailed sample information including GPS location, dates, trap times and horse fly counts and can be found at https://data.gulfresearchinitiative.org/in the data sets by L.F. Adult tabanid population data and available voucher specimens **(**10.7266/N75718ZM) and Tabanid adult and larval collection data, coastal Louisiana, 2016 (10.7266/N7MS3R4Z). Raw data of microsatellite genotypes are available at https://data.gulfresearchinitiative.org/ in the data sets by C.H. *Tabanus nigrovittatus* microsatellite data for the assessment of population genetics of 13 populations sampled from coastal Louisiana, 2010-2011 **(**10.7266/N71J97N2) and *Tabanus nigrovittatus* microsatellite data for the assessment of population genetics of populations sampled from coastal Louisiana, 2012–2017 (10.7266/N7MK6B8V).

## Electronic supplementary material

Supplementary Information
