# Supplementary material for: Recovery of horse fly populations in Louisiana marshes following the Deepwater Horizon oil spill
Source: Sci Rep. 2018 Sep 13;8:13777. doi: 10.1038/s41598-018-31442-1 (PMC6137066; doi:10.1038/s41598-018-31442-1)

Supplementary Information

Recovery of horse fly populations in Louisiana marshes following the Deepwater Horizon oil spill

CLAUDIA HUSSENEDER[1*], JONG-SEOK PARK[1, 2], AND LANE D. FOIL[1]

[1] *Department of Entomology, Louisiana State University Agricultural Center, Baton Rouge, LA 70803*

[2] *S1-5 204b, Chungbuk National University, 1Chungdae-ro, Seowon-gu, Chengju, Chungbuk, 28644, South Korea*

Table S1: Mean genetic distances (F_{ST}) among populations from non-oiled and oiled regions and between populations from non-oiled and oiled regions.

Different letters within the same row indicate statistical significance in genetic distances between years (two-tailed difference of means test, 300 permutations, $P \leq 0.05$). Different letters within the same column indicate significant differences when comparing genetic distances among populations from non-oiled and oiled regions and between populations from non-oiled and oiled regions. If any of the letters are shared, the difference is not significant.

n/a: SD could not be calculated because the 2015 data set contained only two non-oiled control populations.

**Populations**	**2010**		**2011**		**2015**		**2016**	
**non-oiled**	0.061	a	0.243	ac	0.220	ad	0.207	ace
SD	0.013		0.138		n/a		0.198	
**oiled**	0.259	b	0.200	ab	0.129	ac	0.083	ce
SD	0.032		0.054		0.113		0.064	
**between non-oiled and oiled**	0.387	c	0.389	cd	0.295	d	0.163	e
SD	0.025		0.146		0.091		0.095	

Table S2: Directional migration rates of adult tabanids from and into non-oiled and oiled regions. Different letters indicate significant differences in rate of emigration (a) or immigration (b) within rows (across years) and columns (non-oiled vs oiled in same year). Significances were determined by non-parametric Mann Whitney U tests ($p \leq 0.05$).

**Year**	**2010**		**2011**		**2015**		**2016**	
**(a) Emigration from**								
**Non-oiled**	**0.013**	a	**0.011**	ac	**0.032**	b	**0.031**	b
SD	0.019		0.015		0.014		0.052	
**Oiled**	**0.003**	a	**0.011**	ac	**0.037**	bc	**0.016**	ac
SD	0.0004		0.015		0.071		0.035	
**(b) Immigration into**								
**Non-oiled**	**0.018**	ab	**0.010**	b	**0.003**	ab	**0.019**	a
	0.015		0.010		0.000		0.046	
**Oiled**	**0.003**	a	**0.017**	c	**0.056**	bcd	**0.036**	d
	0.003		0.010		0.072		0.047	

Figure S1: Determining the number of genetic clusters (Kmax) most representative to the 2010-2016) tabanid genotype data set.

Deviance information criterion (DIC) values from 26 potential Kmax (10 runs each) were plotted and the number of genetic clusters was set at the value where DIC stabilized and the curve flattened out (Kmax=8).

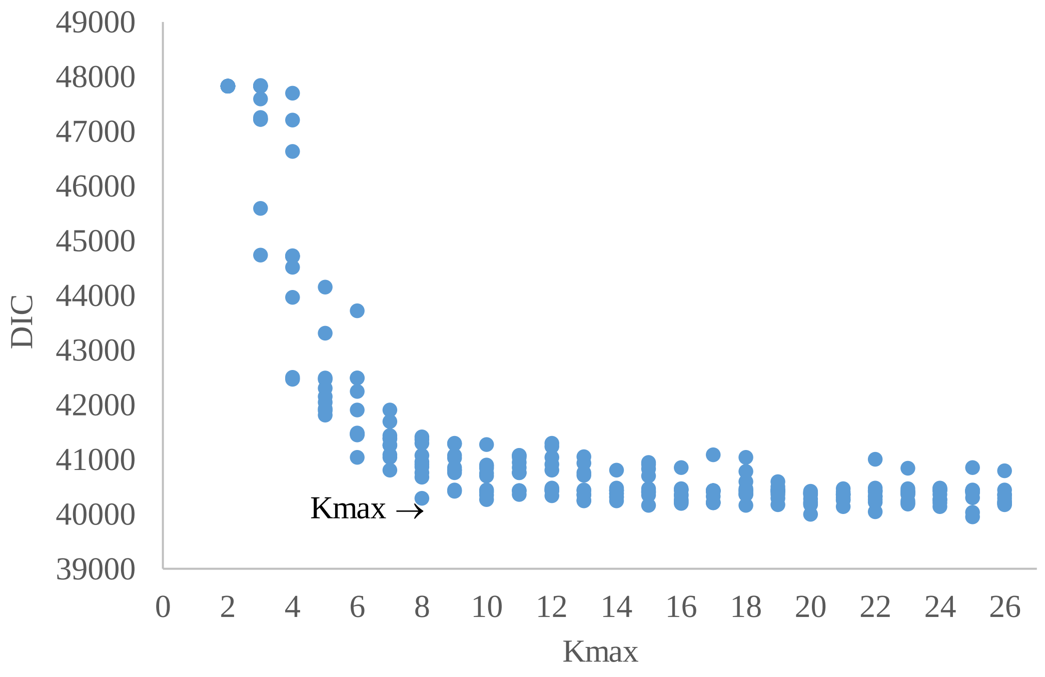

Supplement: Supplementary file 1 — Supplementary Information [file 41598_2018_31442_MOESM1_ESM.pdf]
